# Association of per-and polyfluoroalkyl substances with thyroid hormones in the umbilical cord blood of neonates born by spontaneous delivery

**DOI:** 10.3389/fpubh.2025.1528588

**Published:** 2025-04-02

**Authors:** Yubo Song, Wei Du, Jufeng Hu

**Affiliations:** Department of Obstetrics, Women and Children Health Care Hospital of Linyi, Linyi, China

**Keywords:** umbilical cord blood, neonates, spontaneous delivery, per-and polyfluoroalkyl substances, thyroid hormones

## Abstract

**Objective:**

Per-and polyfluoroalkyl substances (PFASs) affect thyroid function, impairing neonatal development and growth. This study aims to explore the association between PFASs and thyroid hormones in the umbilical cord blood of neonates delivered spontaneously.

**Methods:**

A total of 119 puerperae who delivered vaginally were included. Twenty-nine PFASs were quantified in the umbilical cord plasma using a Waters ACQUITY ultra-performance liquid chromatography (UPLC) system coupled with a Waters Quattro Premier XE triple quadrupole mass spectrometer. Five thyroid hormones were quantified in umbilical cord plasma using a Roche Analytics E170 modular analyzer.

**Results:**

Perfluorooctanoic acid (PFOA), 6:2 chlorinated polyfluorinated ether sulfonic acid (6:2 Cl-PFESA), and linear perfluorooctane sulfonic acid (L-PFOS) were present in the highest levels in the umbilical cord blood with median (quartile 1–quartile 3) levels of 3.23 (2.32–4.32), 1.35 (0.84–2.01), and 0.94 (0.63–1.41) ng/mL, respectively. The linear regression analysis revealed that linear perfluorohexane sulfonic acid (L-PFHxS) (*β* = 0.557, *p* = 0.038) and perfluorononanoic acid (PFNA) (β = 0.613, *p* = 0.045) were independently and positively associated with free triiodothyronine (T3), but PFOA exhibited an inverse trend (*β* = −0.040, *p* = 0.002). The sum of 3,4,5 monohydroperfluorooctane sulfonates (Σ3,4,5 m-PFOS) was independently and negatively associated with total T3 (*β* = −0.349, *p* = 0.007). Perfluorododecanoic acid (PFDoA) was found to have a positively correlation with total T3 (*β* = 2.107, *p* = 0.027) and free T3 (β = 5.254, *p* = 0.008).

**Conclusion:**

L-PFHxS, PFNA, PFOA, Σ3,4,5 m-PFOS, and PFDoA are associated with thyroid hormones in the umbilical cord blood of neonates delivered spontaneously.

## 1 Introduction

Thyroid hormones play an important role in neonatal development (1). The release of thyroid-stimulating hormone (TSH) triggers thyroxine (T4) secretion, which is subsequently converted into the more active form, triiodothyronine (T3) (2–4). Most of the T4 and T3 bind to transport proteins in the bloodstream, while free T3 (FT3) and free T4 (FT4) are unbound, biologically active fractions that can be directly absorbed by cells (2–4). The dysregulation of these thyroid hormones adversely affects cardiovascular systems, bone growth, and neurodevelopment, impairing neonatal growth (2, 5–7). Therefore, exploring the factors associated with thyroid hormone levels is essential for developing corresponding interventions to prevent the impairment of neonatal growth.

Per-and polyfluoroalkyl substances (PFASs) are widely utilized in various commercial products such as food packaging, cookware, lubricants, and clothing (8). It is well recognized that PFASs can interfere with thyroid hormone homeostasis (9, 10). Several studies have explored the association between PFASs and thyroid hormones in pregnant women and adults (11–15). For instance, a previous study found that perfluorononanoic acid (PFNA), perfluorodecanoic acid (PFDA), perfluoroheptanoic acid (PFHpA), perfluorooctanoic acid (PFOA), perfluoroundecanoic acid (PFUnDA), and perfluorooctane sulfonate (PFOS) were associated with thyroid hormones in the serum of pregnant women (11). Another study found that PFUnDA, perfluoroheptane sulfonic acid (PFHpS), and 6:2 chlorinated polyfluorinated ether sulfonic acid (6:2 Cl-PFESA) were associated with thyroid hormones in the serum of Chinese adults (12). In addition, previous studies have shown an association between PFASs and thyroid hormones in the umbilical cord blood (16–20). For instance, PFOS was found to be negatively associated with T4 but positively associated with TSH in the umbilical cord blood (16). Another study found that several PFASs, such as PFOS, PFNA, PFDA, perfluoroundecanoic acid (PFUnDA), and perfluorododecanoic acid (PFDoA), were associated with thyroid hormones in the umbilical cord blood (17). Previous studies analyzed a small number of PFASs (≤10) (16–20). Furthermore, thyroid hormone levels can be affected by cesarean or vaginal delivery (21). Nevertheless, the majority of the previous studies did not analyze this factor, but they enrolled neonates born by both types of delivery (16, 18–20). Although a previous study explored the association between PFASs and thyroid hormones in the umbilical cord blood collected after cesarean delivery (17), evidence from the umbilical cord blood collected after vaginal delivery was scarce.

Accordingly, the present study intended to explore the association between a broader range of PFASs and thyroid hormones in the umbilical cord blood of neonates born by spontaneous delivery.

## 2 Methods

### 2.1 Subjects

The study used the data from a large birth cohort study conducted at the Women and Children’s Healthcare Hospital of Linyi, China. The inclusion and exclusion criteria were detailed in our previous study (22). A total of 420 puerperae were enrolled from January 2019 to December 2021. Among them, 265 puerperae with cesarean delivery and 35 puerperae with thyroid diseases were excluded. A puerpera without complete data on PFASs was also excluded. Thus, 119 puerperae who delivered vaginally at 36–42 weeks of gestation were included in this study. The Ethics Committee of our hospital approved this study (approval number: KYL-YXLL-2021015). Informed consent was obtained from all participants.

### 2.2 Data collection and sample processing

The clinical characteristics of puerperae, including age, height, weight, body mass index (BMI), education level, times of deliveries, and gestational weeks, were collected. The birth height and birth weight of neonates were also collected.

After vaginal delivery, the umbilical cord blood samples were collected by a trained nurse using disposable syringes under sterile conditions. Then, the samples were centrifuged. The plasma was separated and aliquoted into polypropylene EP tubes using a disposable sterile Pasteur pipette. The umbilical cord plasma was stored at −80°C until chemical analysis.

### 2.3 PFAS quantification

A total of 29 PFASs were quantified in the umbilical cord plasma using a Waters ACQUITY ultra-performance liquid chromatography (UPLC) system (Waters Corporation, Milford, MA) coupled with a Waters Quattro Premier XE triple quadrupole mass spectrometer: 6:2 Cl-PFESA, perfluorobutanoic acid (PFBA), PFHpS, linear perfluorohexane sulfonic acid (L-PFHxS), PFNA, PFOA, linear PFOS (L-PFOS), sum of 3,4,5 monohydroperfluorooctane sulfonates (Σ3,4,5 m-PFOS), PFUnDA, PFDA, 6-monohydroperfluorooctane sulfonate (6 m-PFOS), perfluorohexanoic acid (PFHxA), PFDoA, perfluorotetradecanoic acid (PFTeDA), 1-monohydroperfluorooctane sulfonate (1 m-PFOS), PFHpA, perfluorobutane sulfonate (PFBS), hexafluoropropylene oxide dimer acid (HFPO-DA), perfluoropentanoic acid (PFPeA), 4:2 fluorotelomer sulfonic acid (4:2 FTS), perfluorooctane sulfonamide iodide (FOSA-I), sodium dodecafluoro-3 h-4,8-dioxanonanoate (NaDONA), perfluoropentane sulfonic acid (PFPeS), sum of dihydroperfluorooctane sulfonates (Σm2-PFOS), 8:2 fluorotelomer sulfonic acid (8:2 FTS), N-methyl perfluorooctane sulfonamidoacetic acid (N-MeFOSAA), 6:2 fluorotelomer sulfonic acid (6,2 FTS), perfluorononane sulfonic acid (PFNS), and N-ethyl perfluorooctane sulfonamidoacetic acid (N-EtFOSAA).

The Waters ACQUITYTM BEH C18 column (50 mm × 2.1 mm, 1.7 μm, Waters, United States) was used for quantification under the following conditions: column temperature was 40°C, flow rate was 0.4 mL/min, injection volume was 10 μL, and injection mode was partial quantification loop mode. The mobile phase A was 2 mmol/L of ammonium acetate aqueous solution, while the mobile phase B was methanol. The gradient elution was divided into the following stages: 0.0–0.5 min, with 40% of phase B; 0.5–12 min, phase B increased from 40 to 90%; 12–14 min, phase B decreased from 90 to 40%; and 14–15.5 min, held at 40% of phase B. Mass spectrometry was performed in the electrospray ionization (ESI) negative ionization mode with a capillary voltage of 3.0 kV, a source temperature of 120°C, a desolvation temperature of 400°C, a desolvation gas flow rate of 800 L/h, a cone-hole gas flow of 150 L/h, and a collision gas flow rate of 0.15 mL/min. Multiple response monitoring (MRM) mode was used for quantitative analysis.

For each batch of 20 samples, two blanks (Milli-Q pure water) were added to monitor the background during the pretreatment process. Before analyzing the samples, methanol was injected to monitor the instrument background. The detection limit of this method was 0.1–5.0 pg./mL, the limit of quantification was 0.2–10.0 pg./mL, and the recovery rate ranged from 70 to 130%.

### 2.4 Thyroid hormone quantification

A total of five thyroid hormones were quantified in the umbilical cord plasma using a Roche Analytics E170 modular analyzer (Roche Diagnostics, Mannheim, Germany): total T3 (TT3), FT3, total T4 (TT4), FT4, and TSH. The intra-assay coefficients of variation were less than 10%, while the inter-assay coefficients of variation were all below 15%.

### 2.5 Statistics

SPSS version 29.0 (IBM, United States) and R version 4.3.3 were used for data analysis. The clinical characteristics of subjects were described as mean ± standard deviation or numbers with percentage. The Spearman test was used for correlation analyses among PFASs and to determine the correlation between PFASs and thyroid hormones. Due to the high collinearity between PFASs, dimension reduction was completed for the 29 PFASs by elastic network regression. A 20-fold cross-validation method was used, and the loss was calculated using mean-squared error. As a result of dimension reduction, the 29 PFASs were reduced to 9. Furthermore, to explore the independent association between PFASs and thyroid hormones, linear regression models were constructed. The results of dimension reduction and significant results of the Spearman test were included in the model, where the enter method was used. A *p*-value of <0.050 was indicated as statistical significance.

### 2.6 Study design and analytical workflow

Initially, 420 puerperae were included, and 300 of them were excluded because they had a cesarean delivery (*n* = 265) and had thyroid diseases (*n* = 35). A total of 119 puerperae were enrolled, and their umbilical cord plasma samples were collected. Twenty-nine PFASs and five thyroid hormones were quantified. One patient was excluded from the analysis due to incomplete PFAS data. Subsequently, the Spearman test was applied to analyze the correlation between PFASs and thyroid hormones, and elastic network regression was used to deal with multicollinearity among PFASs through dimension reduction. A linear regression model was constructed to explore the independent association between these PFASs and thyroid hormones (Supplementary Figure 1).

## 3 Results

### 3.1 Clinical information

The mean age of puerperae was 28.6 ± 4.6 years. A total of 106 (89.1%) puerperae were primiparity, and the other 13 (10.9%) puerperae were multiparity. The gestational period was 39.3 ± 1.1 weeks. The birth height of neonates was 49.9 ± 0.3 cm, and the birth weight was 3429.1 ± 376.2 g. The detailed clinical information is given in Table 1.

**Table 1:** Clinical characteristics.

Characteristics	Subjects (*N* = 119)
Puerperae	
Age (years), mean ± SD	28.6 ± 4.6
Height (cm), mean ± SD	163.7 ± 4.9
Weight (kg), mean ± SD	59.1 ± 9.9
BMI (kg/m^2^), mean ± SD	22.0 ± 3.4
Education level, *n* (%)	
High school or below	58 (48.7)
Above high school	61 (51.3)
Times of deliveries, *n* (%)	
Primiparity	106 (89.1)
Multiparity	13 (10.9)
Gestational period (weeks), mean ± SD	39.3 ± 1.1
Neonates	
Birth height (cm), mean ± SD	49.9 ± 0.3
Birth weight (g), mean ± SD	3429.1 ± 376.2

SD, standard deviation; BMI, body mass index.

### 3.2 Levels of thyroid hormones and PFASs in the umbilical cord blood

Five thyroid hormones (TT3, FT3, TT4, FT4, and TSH) were quantified in the umbilical cord plasma. The median [quartile 1 (Q1)–quartile 3 (Q3)] levels of TT3, FT3, TT4, FT4, and TSH in the umbilical cord blood were 0.8 (0.7–0.8) nmol/L, 2.1 (2.0–2.3) pmol/L, 98.7 (88.3–111.8) nmol/L, 15.0 (13.8–16.2) pmol/L, and 7.3 (5.7–11.7) μIU/mL, respectively (Table 2).

**Table 2:** Distribution of thyroid hormones in the umbilical cord blood.

Thyroid hormones	Range		Percentiles		
	Minimum	Maximum	25th	50th	75th
TT3 (nmol/L)	0.5	1.1	0.7	0.8	0.8
FT3 (pmol/L)	1.6	2.8	2.0	2.1	2.3
TT4 (nmol/L)	55.4	165.6	88.3	98.7	111.8
FT4 (pmol/L)	11.8	19.6	13.8	15.0	16.2
TSH (μIU/mL)	2.6	39.0	5.7	7.3	11.7

TT3, total triiodothyronine; FT3, free triiodothyronine; TT4, total thyroxine; FT4, free thyroxine; TSH, thyroid stimulating hormone.

A total of 29 PFASs were quantified in the umbilical cord plasma. Nineteen PFASs had a detection rate of >50%: 6:2 Cl-PFESA, PFBA, PFHpS, L-PFHxS, PFNA, PFOA, L-PFOS, Σ3,4,5 m-PFOS, PFUnDA, PFDA, 6 m-PFOS, PFHxA, PFDoA, PFTeDA, 1 m-PFOS, PFHpA, PFBS, HFPO-DA, and PFPeA. Among them, PFOA, 6:2 Cl-PFESA, and L-PFOS possessed the highest levels in the umbilical cord blood, with median (Q1–Q3) levels of 3.23 (2.32–4.32), 1.35 (0.84–2.01), and 0.94 (0.63–1.41) ng/mL, respectively (Table 3).

**Table 3:** Distributions of PFASs in the umbilical cord blood.

PFASs (ng/mL)	Detection rate (%)	Range		Percentiles		
		Minimum	Maximum	25th	50th	75th
6:2 Cl-PFESA	100.0	0.27	8.83	0.84	1.35	2.01
PFBA	100.0	0.01	1.90	0.05	0.08	0.11
PFHpS	100.0	0.00	0.06	0.01	0.02	0.03
L-PFHxS	100.0	0.04	0.65	0.12	0.17	0.22
PFNA	100.0	0.08	0.60	0.18	0.27	0.36
PFOA	100.0	0.96	9.94	2.32	3.23	4.32
L-PFOS	100.0	0.06	3.80	0.63	0.94	1.41
Σ3,4,5 m-PFOS	100.0	0.05	0.70	0.14	0.19	0.27
PFUnDA	99.2	0.00	0.37	0.09	0.12	0.18
PFDA	98.3	0.00	0.42	0.06	0.10	0.14
6 m-PFOS	98.3	0.00	1.04	0.05	0.09	0.17
PFHxA	92.4	0.00	0.07	0.01	0.03	0.04
PFDoA	81.5	0.00	0.08	0.02	0.03	0.04
PFTeDA	81.5	0.00	0.22	0.01	0.02	0.03
1 m-PFOS	77.3	0.00	0.01	0.00	0.00	0.00
PFHpA	61.3	0.00	1.17	0.00	0.03	0.10
PFBS	58.8	0.00	0.16	0.00	0.01	0.02
HFPO-DA	58.0	0.00	0.09	0.00	0.00	0.01
PFPeA	52.9	0.00	0.07	0.00	0.00	0.01
4:2 FTS	16.8	0.00	0.02	0.00	0.00	0.00
FOSA-I	16.0	0.00	0.05	0.00	0.00	0.00
NaDONA	10.1	0.00	0.00	0.00	0.00	0.00
PFPeS	9.2	0.00	0.02	0.00	0.00	0.00
Σm2-PFOS	5.0	0.00	0.04	0.00	0.00	0.00
8:2 FTS	3.4	0.00	0.02	0.00	0.00	0.00
N-MeFOSAA	3.4	0.00	0.01	0.00	0.00	0.00
6:2 FTS	0.8	0.00	0.04	0.00	0.00	0.00
PFNS	0.8	0.00	0.04	0.00	0.00	0.00
N-EtFOSAA	0.0	0.00	0.00	0.00	0.00	0.00

PFASs, per-and polyfluoroalkyl substances; 6:2 Cl-PFESA, 6:2 chlorinated polyfluorinated ether sulfonic acid; PFBA, perfluorobutanoic acid; PFHpS, perfluoroheptane sulfonic acid; L-PFHxS, linear perfluorohexane sulfonic acid; PFNA, perfluorononanoic acid; PFOA, perfluorooctanoic acid; L-PFOS, linear perfluorooctane sulfonic acid; Σ3,4,5 m-PFOS, sum of 3,4,5 monohydroperfluorooctane sulfonates; PFUnDA, perfluoroundecanoic acid; PFDA, perfluorodecanoic acid; 6 m-PFOS, 6-monohydroperfluorooctane sulfonate; PFHxA, perfluorohexanoic acid; PFDoA, perfluorododecanoic acid; PFTeDA, perfluorotetradecanoic acid; 1 m-PFOS, 1-monohydroperfluorooctane sulfonate; PFHpA, perfluoroheptanoic acid; PFBS, perfluorobutane sulfonate; HFPO-DA, hexafluoropropylene oxide dimer acid; PFPeA, perfluoropentanoic acid; 4:2 FTS, 4:2 fluorotelomer sulfonic acid; FOSA-I, perfluorooctane sulfonamide iodide; NaDONA, sodium dodecafluoro-3 h-4,8-dioxanonanoate; PFPeS, perfluoropentane sulfonic acid; Σm2-PFOS, sum of dihydroperfluorooctane sulfonates; 8:2 FTS, 8:2 fluorotelomer sulfonic acid; N-MeFOSAA, N-methyl perfluorooctane sulfonamidoacetic acid; 6:2 FTS, 6:2 fluorotelomer sulfonic acid; PFNS, perfluorononane sulfonic acid; N-EtFOSAA, N-ethyl perfluorooctane sulfonamidoacetic acid.

### 3.3 Correlation between PFASs and thyroid hormones in the umbilical cord blood

To explore the correlation between PFASs and thyroid hormones, the Spearman test was used. It was found that Σ3,4,5 m-PFOS was negatively correlated with TT3 (*r* = −0.184, *p* = 0.045). Furthermore, PFUnDA (*r* = −0.201, *p* = 0.029), 6 m-PFOS (*r* = −0.221, *p* = 0.016), and 4:2 FTS (*r* = −0.192, *p* = 0.036) were negatively correlated with TSH. PFDoA was positively correlated with FT3 (*r* = 0.209, *p* = 0.022) but negatively correlated with TSH (*r* = −0.201, *p* = 0.028). These findings suggested a complex interplay between specific PFASs and thyroid hormone regulation in the context of the umbilical cord blood. No correlations were found between other PFASs and thyroid hormones in the umbilical cord blood (all *p* > 0.050) (Table 4).

**Table 4:** Correlation analyses between PFASs and thyroid hormones by the Spearman test.

PFASs	TT3		FT3		TT4		FT4		TSH	
	*r*	*P*-value	*r*	*P*-value	*r*	*P*-value	*r*	*P*-value	*r*	*P*-value
6:2 Cl-PFESA	−0.125	0.174	−0.087	0.346	−0.092	0.319	−0.058	0.532	−0.079	0.390
PFBA	0.071	0.441	0.060	0.519	0.034	0.712	0.173	0.060	−0.111	0.230
PFHpS	−0.082	0.374	0.009	0.919	−0.081	0.382	−0.070	0.449	−0.127	0.170
L-PFHxS	−0.013	0.887	0.061	0.510	−0.124	0.180	−0.094	0.307	0.005	0.961
PFNA	−0.017	0.857	0.080	0.387	−0.086	0.350	−0.023	0.805	−0.090	0.330
PFOA	−0.097	0.296	−0.033	0.718	−0.093	0.315	0.008	0.935	0.107	0.247
L-PFOS	−0.117	0.206	0.013	0.885	−0.018	0.845	0.032	0.727	−0.128	0.165
Σ3,4,5 m-PFOS	**−0.184**	**0.045**	−0.076	0.409	−0.169	0.067	−0.139	0.130	−0.134	0.145
PFUnDA	−0.049	0.599	0.044	0.638	−0.052	0.574	0.043	0.639	**−0.201**	**0.029**
PFDA	0.002	0.986	0.117	0.204	−0.018	0.847	0.044	0.632	−0.172	0.062
6 m-PFOS	−0.004	0.969	−0.018	0.843	−0.042	0.652	−0.085	0.359	**−0.221**	**0.016**
PFHxA	−0.047	0.613	−0.042	0.653	−0.128	0.164	−0.075	0.417	0.052	0.575
PFDoA	0.097	0.292	**0.209**	**0.022**	0.041	0.661	0.012	0.900	**−0.201**	**0.028**
PFTeDA	0.003	0.974	0.115	0.212	0.037	0.686	0.024	0.798	−0.167	0.070
1 m-PFOS	−0.106	0.252	0.019	0.840	−0.083	0.367	0.005	0.958	−0.104	0.259
PFHpA	−0.026	0.781	−0.095	0.303	−0.173	0.060	−0.054	0.560	0.114	0.218
PFBS	0.044	0.635	−0.056	0.542	−0.048	0.604	0.071	0.444	−0.169	0.066
HFPO-DA	0.078	0.396	0.101	0.274	0.061	0.509	−0.053	0.569	0.071	0.446
PFPeA	−0.055	0.553	0.110	0.234	0.022	0.816	0.010	0.910	0.153	0.097
4:2 FTS	0.177	0.055	0.163	0.077	0.071	0.442	0.073	0.431	**−0.192**	**0.036**
FOSA-I	−0.030	0.746	−0.015	0.873	−0.011	0.909	−0.065	0.483	0.128	0.164
NaDONA	0.079	0.390	0.086	0.350	−0.057	0.539	−0.092	0.320	0.074	0.422
PFPeS	0.008	0.935	0.081	0.381	0.102	0.271	0.122	0.186	−0.056	0.546
Σm2-PFOS	0.011	0.908	−0.087	0.347	0.055	0.551	0.010	0.910	−0.047	0.611
8:2 FTS	0.111	0.231	0.095	0.306	0.014	0.881	0.035	0.709	−0.075	0.419
N-MeFOSAA	0.094	0.311	0.047	0.614	0.120	0.194	0.119	0.197	−0.035	0.705
6:2 FTS	0.123	0.181	0.147	0.110	−0.012	0.896	0.027	0.772	0.056	0.543
PFNS	0.053	0.566	0.072	0.436	0.028	0.763	−0.073	0.431	−0.008	0.932
N-EtFOSAA	0.115	0.213	0.156	0.090	0.025	0.784	−0.032	0.727	−0.005	0.960

PFASs, per-and polyfluoroalkyl substances; TT3, total triiodothyronine; FT3, free triiodothyronine; TT4, total thyroxine; FT4, free thyroxine; TSH, thyroid stimulating hormone; 6:2 Cl-PFESA, 6:2 chlorinated polyfluorinated ether sulfonic acid; PFBA, perfluorobutanoic acid; PFHpS, perfluoroheptane sulfonic acid; L-PFHxS, linear perfluorohexane sulfonic acid; PFNA, perfluorononanoic acid; PFOA, perfluorooctanoic acid; L-PFOS, linear perfluorooctane sulfonic acid; Σ3,4,5 m-PFOS, sum of 3,4,5 monohydroperfluorooctane sulfonates; PFUnDA, perfluoroundecanoic acid; PFDA, perfluorodecanoic acid; 6 m-PFOS, 6-monohydroperfluorooctane sulfonate; PFHxA, perfluorohexanoic acid; PFDoA, perfluorododecanoic acid; PFTeDA, perfluorotetradecanoic acid; 1 m-PFOS, 1-monohydroperfluorooctane sulfonate; PFHpA, perfluoroheptanoic acid; PFBS, perfluorobutane sulfonate; HFPO-DA, hexafluoropropylene oxide dimer acid; PFPeA, perfluoropentanoic acid; 4:2 FTS, 4:2 fluorotelomer sulfonic acid; FOSA-I, perfluorooctane sulfonamide iodide; NaDONA, sodium dodecafluoro-3 h-4,8-dioxanonanoate; PFPeS, perfluoropentane sulfonic acid; Σm2-PFOS, sum of dihydroperfluorooctane sulfonates; 8:2 FTS, 8:2 fluorotelomer sulfonic acid; N-MeFOSAA, N-methyl perfluorooctane sulfonamidoacetic acid; 6:2 FTS, 6:2 fluorotelomer sulfonic acid; PFNS, perfluorononane sulfonic acid; N-EtFOSAA, N-ethyl perfluorooctane sulfonamidoacetic acid. The bold values indicated statistically significant results for the correlation between PFASs and thyroid hormones.

### 3.4 Correlation analysis and dimension reduction among PFASs in the umbilical cord blood

**Figure 1:**
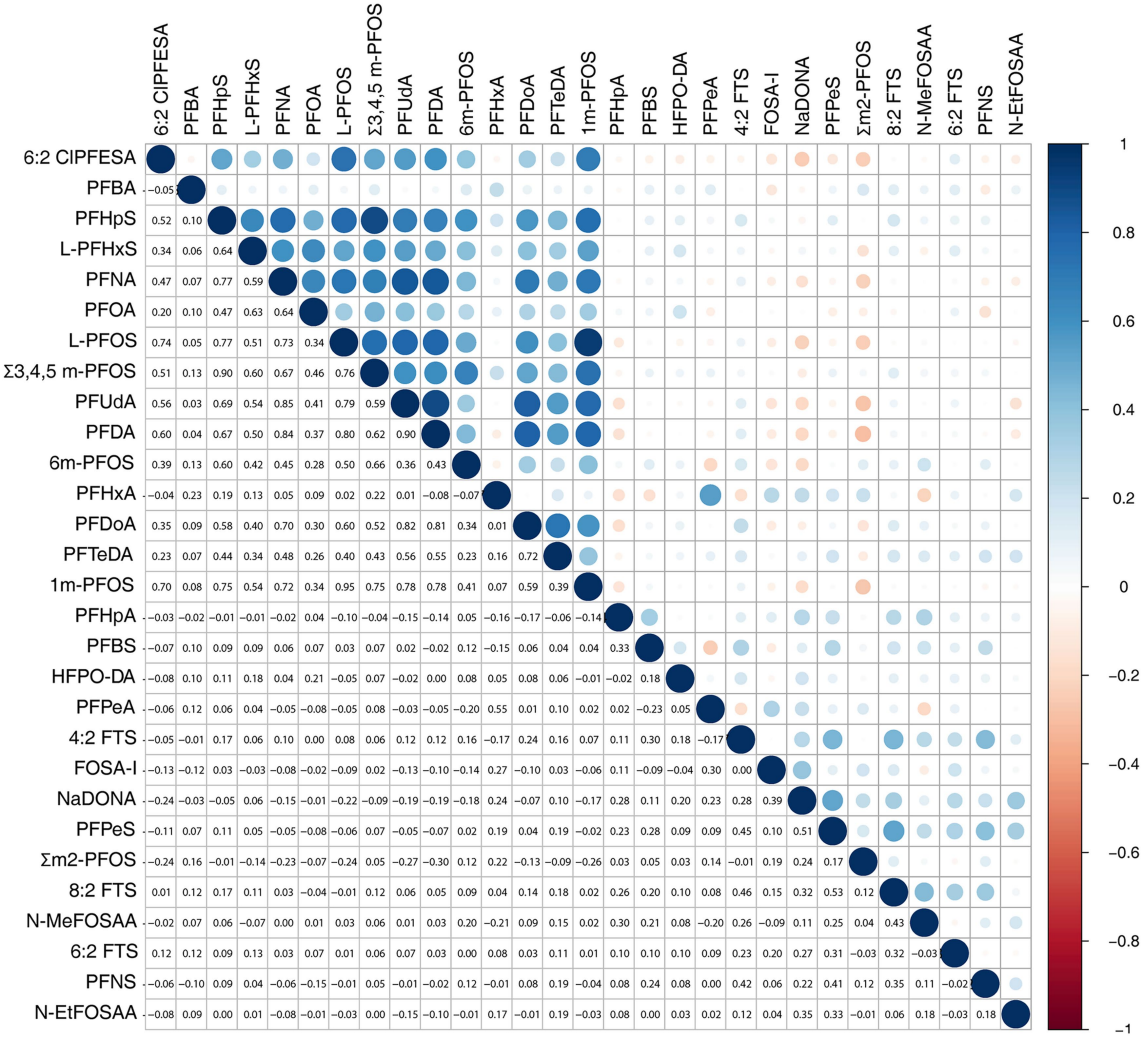
Correlation among PFASs in the umbilical cord blood by the Spearman test.

To explore the relationships among PFASs in the umbilical cord blood, we generated a correlation heatmap that revealed intercorrelations among these compounds (Figure 1). These intercorrelations might introduce multicollinearity, which can complicate the interpretation of statistical models. To address this issue, we used elastic network regression, a technique that combines the strengths of LASSO and ridge regression, to perform dimension reduction on 29 PFASs. Subsequently, nine PFASs were screened out: 6:2 Cl-PFESA, PFBA, L-PFHxS, PFNA, PFOA, 6 m-PFOS, PFHxA, PFTeDA, and N-MeFOSAA (Supplementary Figure 2).

### 3.5 Independent association between PFASs and thyroid hormones in the umbilical cord blood

To further elucidate the association between PFASs and thyroid hormones in the umbilical cord blood, we conducted an analysis using linear regression models. Given that the results from elastic network regression did not include all five PFASs that were correlated with thyroid hormones in the Spearman analysis, and to maximize the inclusion of PFASs, we decided to incorporate the results obtained from both the Spearman analysis and the elastic network regression into the construction of linear regression models.

A total of 13 PFASs were enrolled in linear regression analysis: 6:2 Cl-PFESA, PFBA, L-PFHxS, PFNA, PFOA, Σ3,4,5 m-PFOS, PFUnDA, 6 m-PFOS, PFHxA, PFDoA, PFTeDA, 4:2 FTS, and N-MeFOSAA. L-PFHxS (*β* = 0.557, *p* = 0.038) and PFNA (*β* = 0.613, *p* = 0.045) were independently and positively associated with FT3. However, PFOA was independently and negatively associated with FT3 (*β* = −0.040, *p* = 0.002). Σ3,4,5 m-PFOS was independently and negatively associated with TT3 (β = −0.349, *p* = 0.007). PFDoA was independently and positively associated with TT3 (β = 2.107, *p* = 0.027) and FT3 (β = 5.254, *p* = 0.008). These findings indicated that there was a strong interaction between these five PFASs and thyroid hormones in the umbilical cord blood. No associations were found between other PFASs and thyroid hormones in the umbilical cord blood (all *p* > 0.050) (Table 5).

**Table 5:** Association between PFASs and thyroid hormones by linear regression models.

PFASs	TT3		FT3		TT4		FT4		TSH	
	β (95% CI)	*P*-value	β (95% CI)	*P*-value	β (95% CI)	*P*-value	β (95% CI)	*P*-value	β (95% CI)	*P*-value
6:2 Cl-PFESA	−0.011 (−0.029 to 0.008)	0.267	−0.028 (−0.067 to 0.011)	0.159	−2.182 (−5.750 to 1.385)	0.228	−0.173 (−0.478 to 0.131)	0.261	0.265 (−0.942 to 1.472)	0.664
PFBA	0.059 (−0.023 to 0.140)	0.157	0.088 (−0.082 to 0.257)	0.307	−7.088 (−22.552 to 8.377)	0.366	−0.259 (−1.578 to 1.060)	0.698	3.212 (−2.019 to 8.444)	0.226
L-PFHxS	0.247 (−0.006 to 0.500)	0.055	0.557 (0.032 to 1.083)	**0.038**	−11.057 (−59.087 to 36.974)	0.649	−2.437 (−6.532 to 1.659)	0.241	2.191 (−14.057 to 18.439)	0.790
PFNA	0.200 (−0.088 to 0.488)	0.171	0.613 (0.014 to 1.212)	**0.045**	−23.126 (−77.863 to 31.611)	0.404	−2.112 (−6.780 to 2.555)	0.372	4.981 (−13.536 to 23.497)	0.595
PFOA	−0.014 (−0.031 to 0.002)	0.084	−0.040 (−0.074 to −0.006)	**0.002**	−0.751 (−3.848 to 2.347)	0.632	0.065 (−0.199 to 0.329)	0.626	0.223 (−0.825 to 1.271)	0.674
Σ3,4,5 m-PFOS	−0.349 (−0.601 to −0.096)	**0.007**	−0.287 (−0.811 to 0.237)	0.280	27.403 (−20.515 to 75.320)	0.259	0.787 (−3.299 to 4.873)	0.703	−0.928 (−17.137 to 15.282)	0.910
PFUnDA	−0.304 (−0.937 to 0.329)	0.343	−1.123 (−2.438 to 0.192)	0.093	14.025 (−106.179 to 134.230)	0.817	6.874 (−3.375 to 17.124)	0.186	−26.433 (−67.096 to 14.230)	0.200
6 m-PFOS	0.166 (−0.004 to 0.335)	0.055	0.079 (−0.273 to 0.432)	0.656	1.603 (−30.616 to 33.821)	0.922	0.377 (−2.370 to 3.124)	0.786	−4.225 (−15.124 to 6.674)	0.444
PFHxA	0.353 (−0.744 to 1.451)	0.525	0.242 (−2.041 to 2.524)	0.834	−193.285 (−401.887 to 15.316)	0.069	−6.216 (−24.003 to 11.572)	0.490	13.297 (−57.269 to 83.864)	0.709
PFDoA	2.107 (0.240 to 3.973)	**0.027**	5.254 (1.374 to 9.134)	**0.008**	20.063 (−334.525 to 374.651)	0.911	−8.227 (−38.463 to 22.009)	0.591	61.139 (−58.812 to 181.090)	0.315
PFTeDA	−0.715 (−1.759 to 0.328)	0.177	−1.019 (−3.188 to 1.150)	0.354	97.065 (−101.170 to 295.301)	0.334	4.516 (−12.387 to 21.420)	0.597	−21.884 (−88.944 to 45.176)	0.519
4:2 FTS	3.099 (−5.316 to 11.514)	0.467	0.481 (−17.013 to 17.976)	0.957	−768.720 (−2367.589 to 830.149)	0.343	−31.950 (−168.286 to 104.386)	0.643	−246.240 (−787.112 to 294.631)	0.369
N-MeFOSAA	10.281 (−11.445 to 32.008)	0.350	21.191 (−23.981 to 66.363)	0.354	3923.211 (−205.166 to 8051.588)	0.062	203.938 (−148.090 to 555.965)	0.253	−53.182 (−1449.745 to 1343.38)	0.940

PFASs, per-and polyfluoroalkyl substances; TT3, total triiodothyronine; FT3, free triiodothyronine; TT4, total thyroxine; FT4, free thyroxine; TSH, thyroid stimulating hormone; CI, confidence interval; 6:2 Cl-PFESA, 6:2 chlorinated polyfluorinated ether sulfonic acid; PFBA, perfluorobutanoic acid; L-PFHxS, linear perfluorohexane sulfonic acid; PFNA, perfluorononanoic acid; PFOA, perfluorooctanoic acid; Σ3,4,5 m-PFOS, sum of 3,4,5 monohydroperfluorooctane sulfonates; PFUnDA, perfluoroundecanoic acid; 6 m-PFOS, 6-monohydroperfluorooctane sulfonate; PFHxA, perfluorohexanoic acid; PFDoA, perfluorododecanoic acid; PFTeDA, perfluorotetradecanoic acid; 4:2 FTS, 4:2 fluorotelomer sulfonic acid; N-MeFOSAA, N-methyl perfluorooctane sulfonamidoacetic acid. The bold values indicated statistically significant results for the independent association between PFASs and thyroid hormones.

## 4 Discussion

PFOS, PFOA, PFNA, PFDA, PFUA, PFHxS, PFDoA, and PFBS can be frequently detected in the umbilical cord blood, according to previous studies (16, 17, 23–25). In the current study, we detected 29 PFASs in the umbilical cord blood and discovered that 19 PFASs had a detection rate of >50%: 6:2 Cl-PFESA, PFBA, PFHpS, L-PFHxS, PFNA, PFOA, L-PFOS, Σ3,4,5 m-PFOS, PFUnDA, PFDA, 6 m-PFOS, PFHxA, PFDoA, PFTeDA, 1 m-PFOS, PFHpA, PFBS, HFPO-DA, and PFPeA. The majority of the frequently detected PFASs in our study were in line with the previous studies (16, 17, 23–25). In addition, 6:2 Cl-PFESA and HFPO-DA were also identified in the umbilical cord blood in our study, which were rarely reported in previous studies (16, 17, 23–25). They serve as alternatives to traditional PFASs (26). In detail, 6:2 Cl-PFESA is widely used in the chromium plating industry, while HFPO-DA is primarily applied in fluorochemical production plants (26). Our findings suggested that relevant industries should control the emission and production of 6:2 Cl-PFESA and HFPO-DA in order to reduce the exposure to these PFASs.

The PFAS levels in the umbilical cord blood vary across different regions (16, 17, 23–25). For instance, in Shanghai, a previous study found that PFOA, PFOS, and PFNA possessed the highest levels in the umbilical cord blood, with median levels of 7.57, 2.51, and 0.66 ng/mL, respectively (17). In Zhoushan, another previous study found that PFOA possessed the highest level, followed by 4:2 FTS and perfluoro-n-tridecanoic acid (PFTrDA) in the umbilical cord blood, with median levels of 1.84, 1.70, and 1.61 ng/mL, respectively (23). In Laizhou Wan, PFOA, PFOS, and PFNA were present in the highest levels in the umbilical cord blood, and their median levels were 34.67, 1.39, and 0.44 ng/mL, respectively (24). In this study, we found that in Linyi, PFOA, 6:2 Cl-PFESA, and L-PFOS possessed the highest levels in the umbilical cord blood, with median levels of 3.23, 1.35, and 0.94 ng/mL, respectively. Overall, in different regions, PFOA and PFOS possessed high levels in the umbilical cord blood, suggesting the wide exposure of pregnant women to PFOA and PFOS-containing products, such as cookware, waterproof clothing, and food containers. However, we also found that 6:2 Cl-PFESA possessed a high level in the umbilical cord blood. 6:2 Cl-PFESA is widely used in chromium plating, textiles, and electronic products (27). Our findings indicated that 6:2 Cl-PFESA emissions by these industries might be substantial in Linyi, leading to high exposure of neonates to 6:2 Cl-PFESA.

PFASs possess thyroid-disrupting effects through various mechanisms, such as binding to transthyretin, activating the NA^+^/K^+^-dependent transport of I^−^, binding to the sites of deiodinases, and inducing transcriptional changes in thyroid-regulating genes (28–30). In the current study, we initially applied the Spearman test to explore the correlation between PFASs and thyroid hormones in the umbilical cord blood. It was found that Σ3,4,5 m-PFOS, PFUnDA, 6 m-PFOS, PFDoA, and 4:2 FTS were correlated with thyroid hormones in the umbilical cord blood. However, as reported by a previous study, collinearity existed among PFASs in the umbilical cord blood (23). In line with this previous study (23), we also discovered the intercorrelation among different PFASs in the umbilical cord blood. The existence of collinearity would affect the reliability of our findings. Therefore, we applied elastic network regression to deal with collinearity, which combines ridge regression and LASSO regression (31). After dimension reduction by elastic network regression, 6:2 Cl-PFESA, PFBA, L-PFHxS, PFNA, PFOA, 6 m-PFOS, PFHxA, PFTeDA, and N-MeFOSAA were further screened out. To explore the independent impact of each PFAS on thyroid hormone levels, multivariate linear regression analysis was conducted in our study. It should be clarified that in order to include as many as possible numbers of PFASs while minimizing the influence of collinearity, we incorporated PFASs from both the Spearman test and elastic network regression into the linear regression analysis. L-PFHxS and PFNA were independently and positively associated with FT3, while PFOA exhibited an inverse trend. Σ3,4,5 m-PFOS was independently and negatively associated with TT3. PFDoA was independently and positively associated with TT3 and FT3. Recently published studies found that some PFASs, such as PFOA, PFOS, PFNA, L-PFHxS, and PFUnDA, were associated with thyroid hormones in adults, neonates, and subfertile females (12, 13, 32, 33). Additionally, several meta-analyses also explored the association between PFASs and thyroid hormones, which disclosed that some PFASs, such as PFOA, PFDA, and PFOS, were associated with thyroid hormones in pregnant women, adults, neonates, and adolescents (34–36). Our findings were in line with these previous studies and meta-analyses. The findings of our study provided valuable information that L-PFHxS, PFNA, PFOA, Σ3,4,5 m-PFOS, and PFDoA were strong factors associated with thyroid hormone levels. Relevant authorities should consider implementing measures, such as controlling relevant product use, strengthening public awareness, and promoting the development of safer alternatives, to reduce the exposure of pregnant women and neonates to PFASs in order to protect against thyroid dysfunction.

The present study contained several limitations. (1) This was a single-center study. Thus, selection bias might exist. (2) Follow-up was not performed. Therefore, the long-term effect of exposure to PFASs on the thyroid function of neonates was unknown and should be further explored. (3) L-PFHxS, PFNA, PFOA, Σ3,4,5 m-PFOS, and PFDoA were strong factors associated with thyroid hormone levels. Further *in vitro* and *in vivo* experiments could be considered to explore the detailed mechanism of these PFASs in regulating thyroid hormones.

In conclusion, L-PFHxS, PFNA, PFOA, Σ3,4,5 m-PFOS, and PFDoA are associated with thyroid hormone levels in the umbilical cord blood of neonates born by spontaneous delivery. Corresponding strategies should be developed and implemented to reduce neonates’ exposure to these PFASs to improve their thyroid function, thereby improving neonatal development and growth.

## Data Availability

The original contributions presented in the study are included in the article/Supplementary material, further inquiries can be directed to the corresponding author.
