# Supplementary material for: Association of per-and polyfluoroalkyl substances with thyroid hormones in the umbilical cord blood of neonates born by spontaneous delivery
Source: Front Public Health. 2025 Apr 2;13:1528588. doi: 10.3389/fpubh.2025.1528588 (PMC12000041; doi:10.3389/fpubh.2025.1528588)
Supplement: Supplementary file 1 [file Data_Sheet_1.DOCX]

***Supplementary Materials***

***
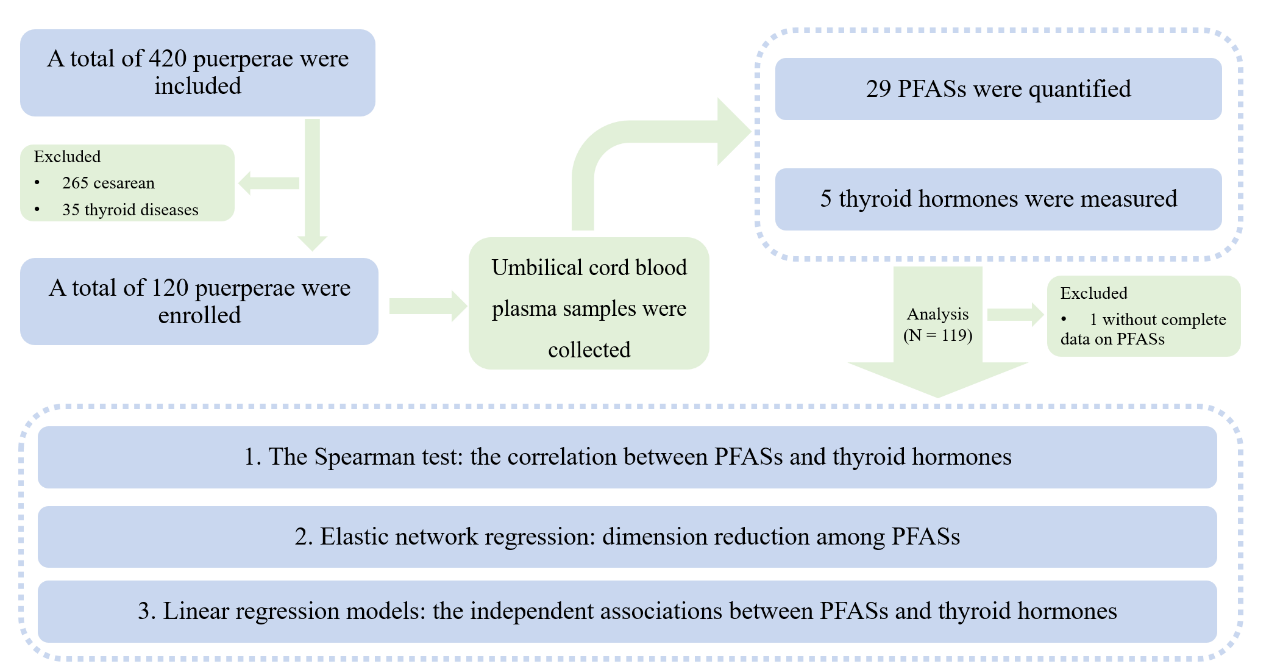
***

**Supplementary Figure 1.** Schematic diagram.


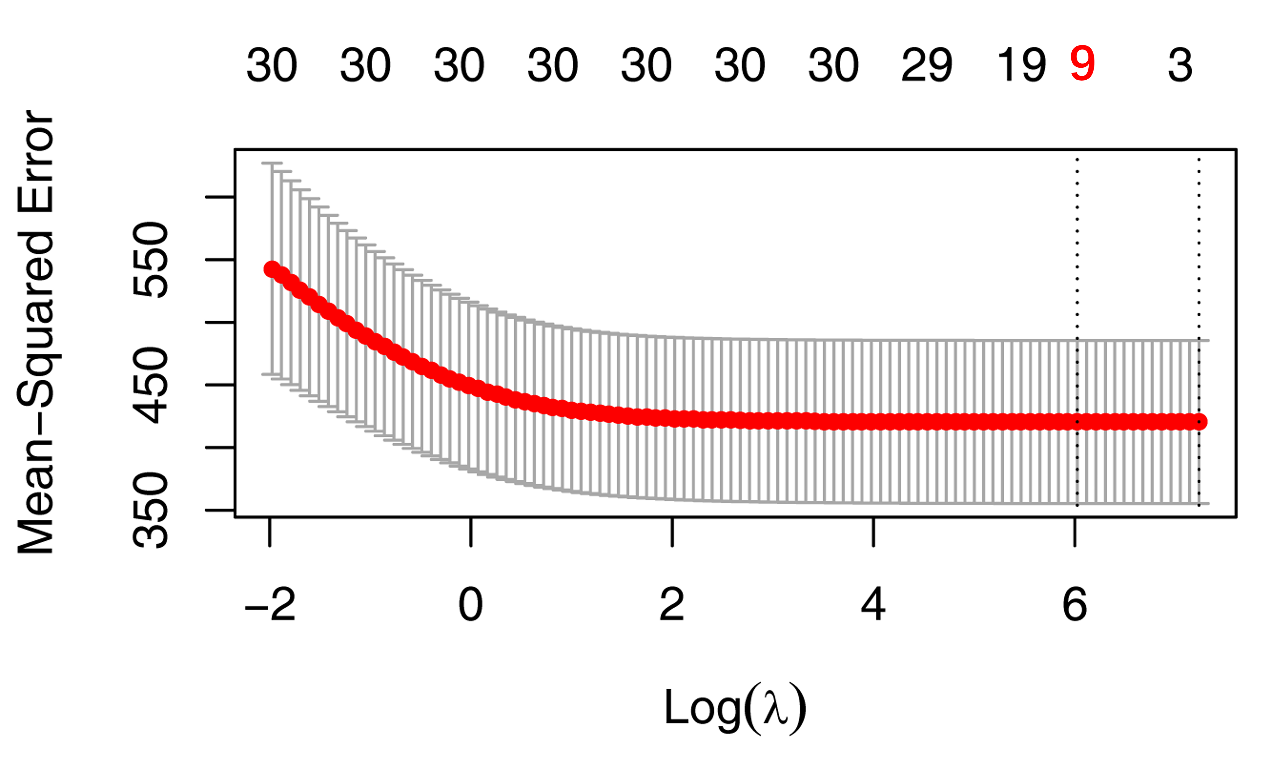


**Supplementary Figure 2.** Dimension reduction of PFASs by elastic network regression.
